# ADAM17/EGFR axis promotes transglutaminase-dependent skin barrier formation through phospholipase C γ1 and protein kinase C pathways

**DOI:** 10.1038/srep39780

**Published:** 2016-12-22

**Authors:** Cristina Wolf, Yawen Qian, Matthew A. Brooke, David P. Kelsell, Claus-Werner Franzke

**Affiliations:** 1 Department of Dermatology, Medical Center - University of Freiburg, Freiburg, Germany; 2 Blizard Institute, Barts and the London School of Medicine and Dentistry, Queen Mary University of London, London, United Kingdom

## Abstract

The vitally important skin barrier is formed by extensive cross-linking activity of transglutaminases (TGs) during terminal epidermal differentiation. We have previously shown that epidermal deficiency of a disintegrin and metalloproteinase 17 (ADAM17), the principal EGFR ligand sheddase, results in postnatal skin barrier defects in mice due to impeded TG activity. However, the mechanism by which ADAM17/EGFR signalling maintains TG activity during epidermal differentiation remains elusive. Here we demonstrate that ADAM17-dependent EGFR signalling promotes TG activity in keratinocytes committed to terminal differentiation by direct induction of TG1 expression. Restored TG1 expression of EGF-stimulated differentiated *Adam17*^*−/−*^ keratinocytes was strongly repressed by inhibitors for PLCγ1 or protein kinase C (PKC) pathways, while treatment with the PKC stimulator 12-*O*-tetradecanoylphorbol-13-acetate restored TG activity in the epidermis of keratinocyte-specific *Adam17*^*−/−*^ (*AD17*^Δ*KC*^) mice. Further investigations emphasized the expression of PKCη, a mediator of *TGM1* transcription, to be sensitive to EGFR activation. In agreement, topical skin application of cholesterol sulfate, an activator of PKCη, significantly improved TG activity in epidermis of *AD17*^Δ*KC*^ mice. Our results suggest ADAM17/EGFR-driven PLCγ1 and PKC pathways as important promoters of TG1 expression during terminal keratinocyte differentiation. These findings may help to identify new therapeutic targets for inflammatory skin diseases related to epidermal barrier defects.

The multilayered epidermis builds up a barrier that protects the body against transepidermal water loss, foreign substances and microbial invasion^1^. This skin barrier is very important for the epidermal homeostasis and needs to be continuously renewed and enzymatically modified^2^. After the basal keratinocytes detach from the underlying basement membrane, they stop to proliferate and become committed to terminal differentiation. During their passage to the skin surface the cells convert into a cornified envelope (CE) that forms the skin barrier. The CE represents an insoluble protein structure that is stabilized by the cross-linking activity of three epidermal transglutaminases (TGs), namely TG1, TG3 and TG5^3^. However, lack of either TG3 or TG5 activity only leads to minor alterations in CE formation and barrier stability^4^,^5^, while lack of TG1 activity causes severe skin barrier defects. The crucial role of TG1 during CE formation is demonstrated in patients with nonsense or missense mutations in the *TGM1* gene, that led to impaired skin barrier formation, transepidermal water loss and skin inflammation in autosomal recessive lamellar ichthyosis or congenital ichthyosiform erythroderma^3^,^6^. However the regulatory mechanisms that control TG activity during skin barrier maintenance remain elusive.

In the epidermis, epidermal growth factor receptor (EGFR) is expressed abundantly in the proliferative basal layer and to a lesser degree in the differentiating suprabasal layers^7^. It is thought that EGFR signalling in basal keratinocytes mainly supports proliferation and survival but prevents differentiation. Moreover it delays apoptosis during early differentiation in suprabasal keratinocytes that have lost their interaction with the matrix^8^,^9^. However, EGFR-ligands are also abundant in differentiated epidermis and there are several evidences that EGFR signalling contributes to terminal keratinocyte differentiation and skin barrier formation^10^,^11^,^12^,^13^. EGFR deficiency causes defects in hair follicle development and immature epidermal differentiation with inflammatory skin reactions in both mice and humans^14^,^15^,^16^,^17^. In addition, EGFR inhibitor therapy in cancer patients commonly induces dermatologic side effects including xerotic itchy skin^18^. Although these data corroborate the relevance of EGFR signalling in skin homeostasis, only little is known about the role of EGFR signalling in skin barrier formation and in suppressing chronic skin inflammation.

ADAM17 (a disintegrin and metalloproteinase 17) or tumor necrosis factor α-converting enzyme (TACE) is a membrane-anchored metalloproteinase that was originally identified to cleave membrane-bound tumor necrosis factor (TNF)-α by a process named as ectodomain shedding^19^. This protease is also known as crucial upstream regulator of EGFR signalling by shedding of the majority of EGFR ligands^20^. Mice lacking ADAM17 die at birth due to defects in heart development and show epithelial abnormalities in several organs, such as intestine and skin^21^. Thereby, *Adam17*^*−/−*^ mice nearly phenocopy mice lacking EGFR, or mice lacking the EGFR ligands TGF-α, HB-EGF, or amphiregulin (AREG), indicating an *in vivo* relevance of ADAM17 in EGFR signalling^22^.

To investigate the role of ADAM17 and EGFR in skin homeostasis, mice with a conditional keratinocyte-specific deletion were generated. *Adam17*^Δ*KC*^ (*AD17*^Δ*KC*^) mice phenocopy *Egfr*^Δ*KC*^ mice in having an intact skin barrier at birth, but developing a pronounced defect in the skin barrier after the third postnatal week leading to more than 80% lethality. The surviving animals develop chronic dermatitis as adults^13^,^16^,^23^. During the last five years several patients with germline loss of function mutations in *ADAM17* or *EGFR* have been described^17^,^24^,^25^, which developed chronic dermatitis with iterated skin infections, very similar to the phenotype of *AD17*^Δ*KC*^ mice, which suggests similar skin barrier defects^13^,^23^. Our investigations on *AD17*^Δ*KC*^ and *Egfr*^Δ*KC*^ mice revealed that ADAM17/EGFR axis sustained the CE formation and postnatal skin barrier stability by tightly regulation of the expression and proteolytic processing of several CE components, especially by supporting TG activity^13^,^26^. Accordingly, application of EGFR ligand TGF-α to *AD17*^Δ*KC*^ mouse skin restored epidermal barrier integrity by stimulating skin TG activity^13^. However, the ADAM17-driven mechanisms that maintain epidermal TG activity during terminal differentiation are not well understood.

Here we demonstrate that ADAM17-dependent EGFR signalling directly induces TG1 expression in keratinocytes committed to terminal differentiation prominently through phospholipase C γ1 (PLCγ1) and protein kinase C (PKC) pathways. Further investigations identified PKCη expression to be responsive to EGFR activation. In agreement, topical skin application of cholesterol sulfate, an activator of PKCη, significantly improved TG activity in epidermis of *AD17*^Δ*KC*^ mice. These findings will help to uncover novel therapeutic strategies for inflammatory skin diseases related to disrupted EGFR signalling.

## Results

### ADAM17-driven EGFR signalling induces expression and activity of TG1 in terminal differentiating keratinocytes

The TG activity in terminal differentiating keratinocyte is composed of activities derived from TG1, TG3 and TG5^2^. We previously demonstrated that lack of *Adam17* in differentiated murine keratinocytes leads to significantly reduced TG activity and decreased expression of TG1 and TG3^13^ as well as reduced *Tgm5* transcription (data not shown). These findings suggest that ADAM17 controls TG activity by modulation of all epithelial TGs. However, lack of either TG3 or TG5 activity causes, if at all, only minor barrier defects^4^,^5^, while lack or strongly reduced TG1 activity leads to severe barrier defects^3^,^6^ as seen in *AD17*^Δ*KC*^ mice. Therefore, we focused our investigations on TG1 expression and activity.

For the determination of TG1 activity we analyzed skin cryo sections or culture cells on coverslips *in situ* using the biotinylated amine donor substrate monodansylcadaverine in a neutral pH 7.4 buffer system, in which TG3 and TG5 are not active^5^,^27^. It has been previously demonstrated that this *in situ* fluorescence technique can discriminate among the activities of TG1 and TG3/TG5 when performed either at neutral pH 7.4 or at basic pH 8.4, respectively. This based on the fact that the catalytic optimum of TG1 lies between pH 7.4 and pH 8.4, while the catalytic optimum for TG3 and TG5 lies at basic pH 8.4^27^,^28^. To investigate how the ADAM17-dependent EGFR signalling regulates TG activity in keratinocytes committed to differentiation, we used suspension cultures on polyhydroxyethylmethacrylate (polyHEMA)-coated plates^10^. This system mimicks terminal differentiation of suprabasal epidermal keratinocytes, since it disrupts the cell-extracellular matrix interaction, while maintaining cell-cell interactions. Time-resolved analysis of the TG activity in polyHEMA cultured wild type keratinocytes revealed strong activity after 6 h, which was retained for at least 48 h (Supplementary Fig. S1a). This correlated with a strong increase in transcription of the genes encoding for TG1 or involucrin (Supplementary Fig. S1b). Since permanent cell cycle exit and commitment to terminal differentiation of keratinocytes occurred within the first 24 h of suspension culture^29^, we have used 24 h suspension culture in our following experiments.

**Figure 1:**
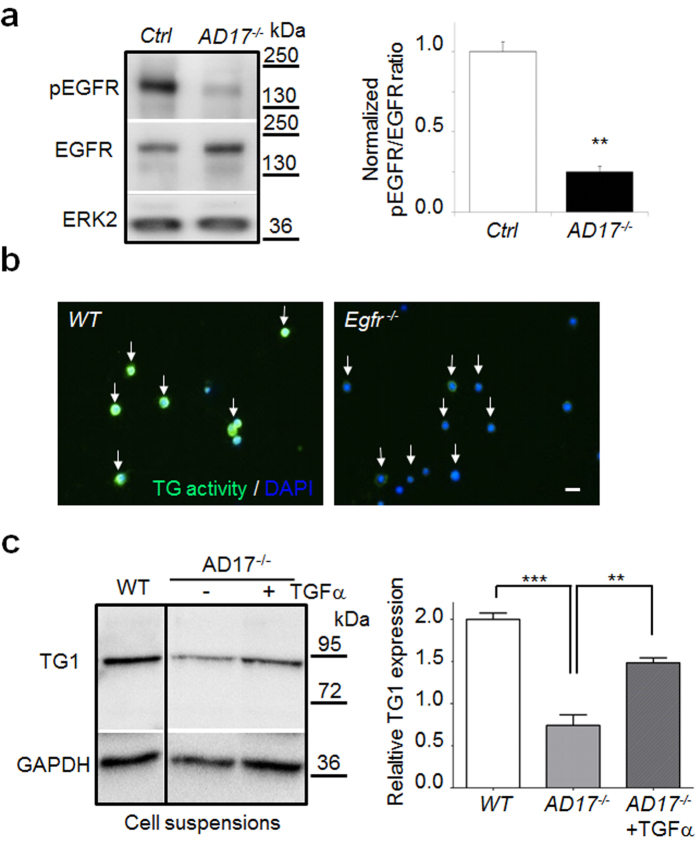
Transglutaminase activity in differentiating mouse keratinocytes is regulated by EGFR signalling. (**a**) WB of primary keratinocyte lysates derived from *AD17*^*∆KC*^ mice or control littermates (Ctrl) revealed significantly reduced level of activated EGFR (normalized ratio of pEGFR/total EGFR), while ERK 2 protein as control was equal. Graph shows band intensities as mean ± SD, n = 3, **p < 0.01. (**b**) Suspension cultured *Egfr*^*−/−*^ keratinocytes analyzed for TG activity at pH 7.4 by fluorescence microscopy revealed strongly reduced signals (white arrows, scale bar, 20 μm) (representative staining from three independent experiments). (**c**) WB analysis of mouse *AD17*^*−/−*^ keratinocytes with or without stimulation with 40 ng/ml TGF-α after 24 h suspension culture with antibodies directed against TG1. (n = 3 per group). Data as mean ± SEM, **p < 0.01, ***p < 0.001.

Analysis of EGFR signalling in *Adam17*^*−/−*^ keratinocytes by Western blot (WB) revealed about 80% reduced EGFR activation (Fig. 1a). To validate whether the reduced TG activity is caused by lack of EGFR signalling, we analyzed 24 h suspension cultures of *Egfr*^*−/−*^ keratinocytes. Indeed, *Egfr*^*−/−*^ keratinocytes showed strongly reduced TG activity (Fig. 1b). In addition, the reduced TG1 expression in ECM-disrupted *Adam17*^*−/−*^ keratinocytes was nearly restored by supplementation with the EGFR-ligand TGF-α (40 ng/ml) on protein (Fig. 1c) and on RNA level (Supplementary Fig. S2), suggesting that the lack of EGFR activation is the major cause of reduced TG1 expression in *Adam17*^*−/−*^ keratinocytes. Since reduced EGFR signalling in the suprabasal *Adam17*^*−/−*^ keratinocytes can lead to decreased survival signals and apoptosis^30^, we analyzed skin sections of 10-day-old *AD17*^Δ*KC*^ mice with skin barrier defects and their littermates by TUNEL labelling. No difference in the proportion of TUNEL-positive keratinocytes was detectable (Supplementary Fig. S3), indicating no increase of apoptosis in the epidermis of *AD17*^Δ*KC*^ mice. Moreover, supplementation of the EGFR-ligands EGF, TGF-α, and Epiregulin (40 ng/ml) to 24 h *Adam17*^*−/−*^ keratinocyte suspension cultures resulted in very similar recovery of TG activity (Supplementary Fig. S4), suggesting coinciding functions among these EGFR-ligands. These results suggest that EGFR signalling accelerates TG activity in differentiating keratinocytes via transcription.

### PKC regulates TG1 expression downstream of EGFR

**Figure 2:**
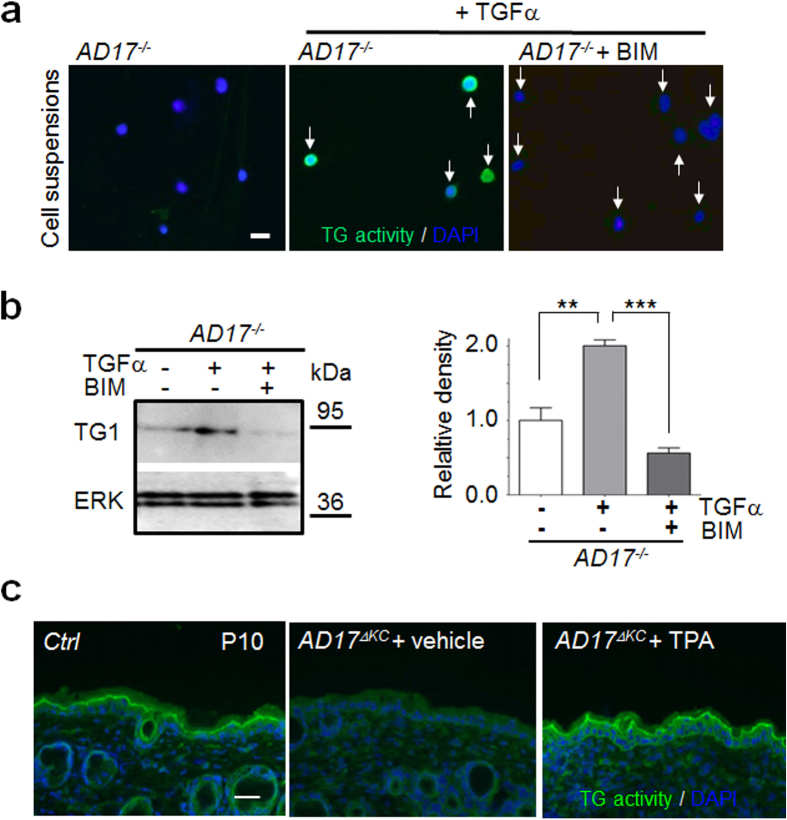
Protein kinase C (PKC) in murine keratinocytes regulates TG1 expression & activity downstream of EGFR *in vitro* and *in vivo*. (**a**,**b**) Mouse wild type and *Adam17*^*−/−*^ keratinocytes were cultured in suspension for 24 h. *Adam17*^*−/−*^ keratinocytes were either treated with 40 ng/ml TGF-α or 40 ng/ml TGF-α and 1 μM bisindolylmaleimide (BIM) and then analyzed by (**a**) *in situ* TG activity detection at pH 7.4 by fluorescence microscopy or (**b**) WB with antibodies against TG1 and ERK as control. The improved TG1 expression and activity of TGF-α stimulated *Adam17*^*−/−*^ keratinocytes was strongly inhibited by 1 μM bisindolylmaleimide. Scale bar, 20 μm. Graph shows band intensities as mean ± SEM, n = 3, **p < 0.01, ***p < 0.001. (**c**) The phorbol ester TPA was topically applied on the shaved skin surface of 10 days old *AD17*^Δ*KC*^ mice (P10) and the skin was analyzed for *in situ* TG activity at pH 7.4 after 6 h. Scale bar, 50 μm. TPA treatment resulted in strongly improved TG activity (representative staining from three independent experiments).

The terminal differentiation of keratinocytes is partly regulated by PKC^31^. These kinases are thought to play an important role in the modulation of transglutaminase activity^32^,^33^,^34^. However, only little is known about the interactions of EGFR and PKC pathways in keratinocytes committed to terminal differentiation. To analyse the interactions of both pathways, we used TGFα-stimulated differentiated *Adam17*^*−/−*^ keratinocytes as a model for EGFR-dependent TG activity. Addition of the general PKC inhibitor bisindolylmaleimide (1 μM) led to strongly reduced TG activity and TG1 expression in TGFα-stimulated differentiated *Adam17*^*−/−*^ keratinocytes (Fig. 2a,b), suggesting a connection of both pathways in the regulation of TG expression and that PKCs are localized downstream of ADAM17/EGFR signalling. These results were confirmed by stimulation experiments with the phorbol ester 12-O-tetradecanoylphorbol-13-acetate (TPA), a strong PKC activator. Stimulation of 24 h suspension cultures with 1 μM TPA completely restored the TG activity in differentiating *Adam17*^*−/−*^ keratinocytes (Supplementary Fig. S5a). WB analysis revealed significantly increased TG1 expression in these cells (Supplementary Fig. S5b), suggesting a transcriptional regulation. To further validate the *in vivo* relevance of our results, we used 10 days old keratinocyte-specific *Adam17*^*−/−*^ mice (A17^ΔKC^) with strongly reduced epidermal TG activity^13^ and topically applied a single dose of 1.5 μg TPA in acetone or acetone vehicle alone (controls) on their back skin. TPA treatment for 6 h resulted in strongly improved TG activity in the epidermis (Fig. 2c). In contrast, TPA treatment for 1 h did not led to improved TG activity (data not shown). In summary, our results demonstrate that the ADAM17/EGFR axis regulates TG1 expression during terminal keratinocyte differentiation via PKCs.

### ADAM17/EGFR axis induces TG activity mainly through PLCγ1 and PKC pathways

**Figure 3:**
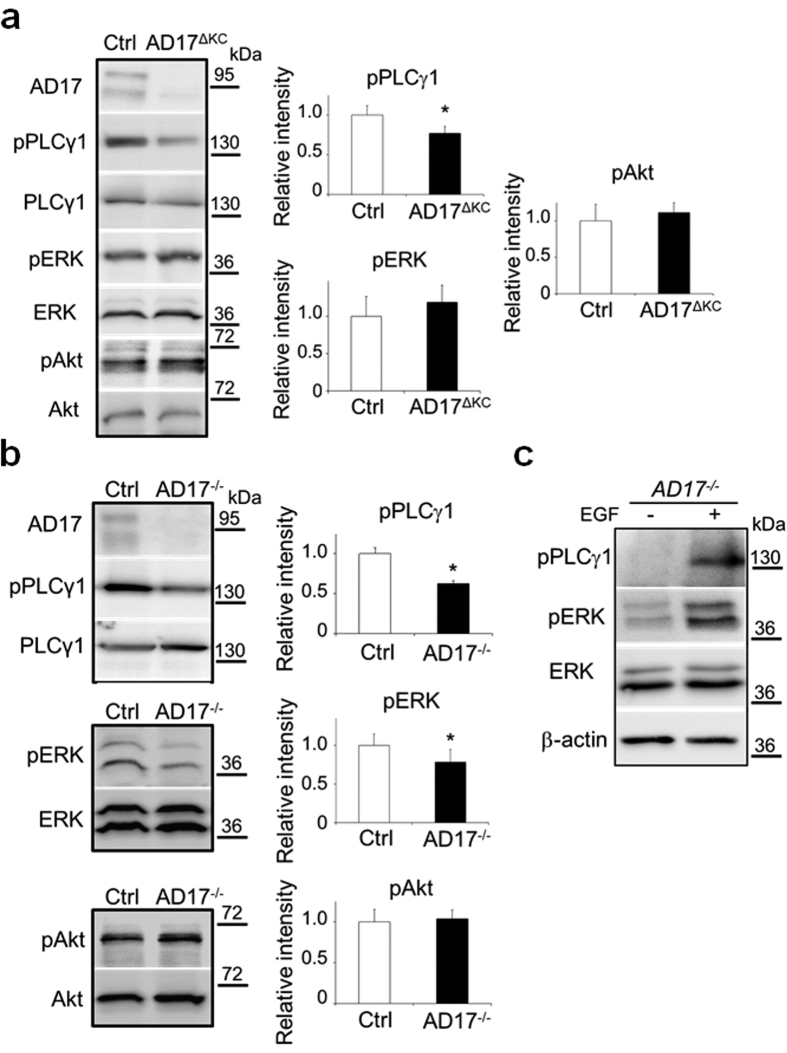
The PLCγ1 pathway is downregulated in murine *Adam17*^*−/−*^ keratinocytes. (**a**,**b**) Representative WBs of (**a**) epidermal splits derived from P10 *AD17*^*∆KC*^ or wild type mice or (**b**) 24 h suspension cultured wild type and *Adam17*^*−/−*^ keratinocytes for activation of PLCγ1, ERK and Akt pathways. The graphs on the right show the mean ± SD of the relative intensities of pPLCγ1/PLCγ1, pERK/ERK and pAkt/Akt (n = 3), *p < 0.05. The activation of the PLCγ1 pathway was significantly reduced in *AD17*^*∆KC*^ epidermis as well as *Adam17*^*−/−*^ keratinocytes. (**c**) After suspension culture for 24 h, *Adam17*^*−/−*^ keratinocytes were treated with 40 ng/ml EGF or vehicle for additional 30 min and analyzed by WB for activation of PLCγ1 and ERK. Both, pPLCγ1 and pERK were strongly increased by EGF stimulation (representative blots of three independent experiments).

**Figure 4:**
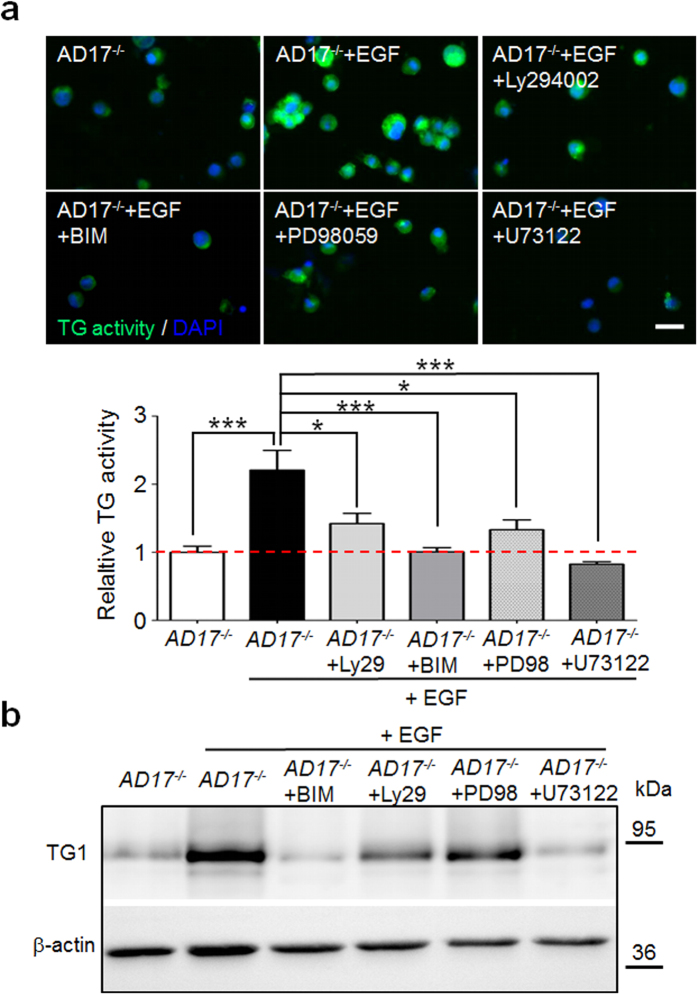
EGFR induced TG activity in murine *Adam17*^*−/−*^ keratinocytes is mainly driven by PLCγ1 and PKC signalling. (**a**) Murine *Adam17*^*−/−*^ keratinocytes were cultured in suspension on poly-HEMA for 24 h in the presence of 40 ng/ml EGF and either DMSO (vehicle control), 50 μM Ly294002, 1 μM bisindolylmaleimide (BIM), 50 μM PD98059, or 5 μM U73122. Afterwards, the keratinocytes were analyzed for TG activity at pH 7.4 by fluorescence microscopy. The addition of either the PLC inhibitor U73122 or the general PKC inhibitor bisindolylmaleimide completely decreased EGF-induced TG activity in *Adam17*^*−/−*^ keratinocytes, while addition of the selective PI3K inhibitor Ly294002 or the selective MEK1 and MEK2 inhibitor PD98059 caused partially inhibition. (n = 4). Scale bar, 20 μm. Graph shows quantitative analysis of fluorescence intensities: mean ± SEM, *p < 0.05, ***p < 0.001. (**b**) WB analysis of cell lysates from the in (**a**) treated *Adam17*^*−/−*^ keratinocytes with antibodies against TG1. β-actin was used as loading control (representative blots of two experiments).

To identify the pathways by which the ADAM17/EGFR axis regulates TG activity in keratinocytes during terminal differentiation we have analyzed the MAPK, PI3K/Akt and PLC/PKC signalling as well known EGFR downstream pathways^9^,^35^. WB analysis of lysates of epidermis splits derived from 10 day old *AD17*^Δ*KC*^ mice with strongly decreased TG activity^13^ revealed significant reduced activation of the PLCγ1 pathway, but no changes in ERK1/2 and Akt activation (Fig. 3a). The reduction in PLCγ1 signalling was verified as a cell-autonomous mechanism, since it was also detected in differentiation-committed *Adam17*^*−/−*^ keratinocytes (Fig. 3b). In addition, we also saw reduced ERK1/2 signalling in these cells (Fig. 3b), suggesting that the ERK pathway might be involved as well. The PLCγ1 and the ERK1/2 signalling was strongly induced by EGF stimulation of 24 h *Adam17*^*−/−*^ keratinocyte suspension cultures, validating the responsiveness of both pathways in differentiation-committed keratinocytes (Fig. 3c). Using EGF-stimulated differentiated *Adam17*^*−/−*^ keratinocytes as a model for EGFR dependent TG activity, the addition of either the PLC inhibitor U73122 or the general PKC inhibitors bisindolylmaleimide or Gö6983 (data not shown) strongly repressed TG activity. However, the addition of the selective PI3K inhibitor Ly294002 or the selective MEK1 and MEK2 inhibitor PD98059 led to partial inhibition of TG activity in these cells (Fig. 4a). Further analysis of TG1 expression of those cells by WB revealed very similar results (Fig. 4b). In conclusion, these results suggest that especially the PLCγ1 and PKC pathways are involved in the regulation of TG activity in differentiation-committed keratinocytes.

### Expression of PKCη, but not PKCδ is responsive to EGFR/PLCγ1 signalling

**Figure 5:**
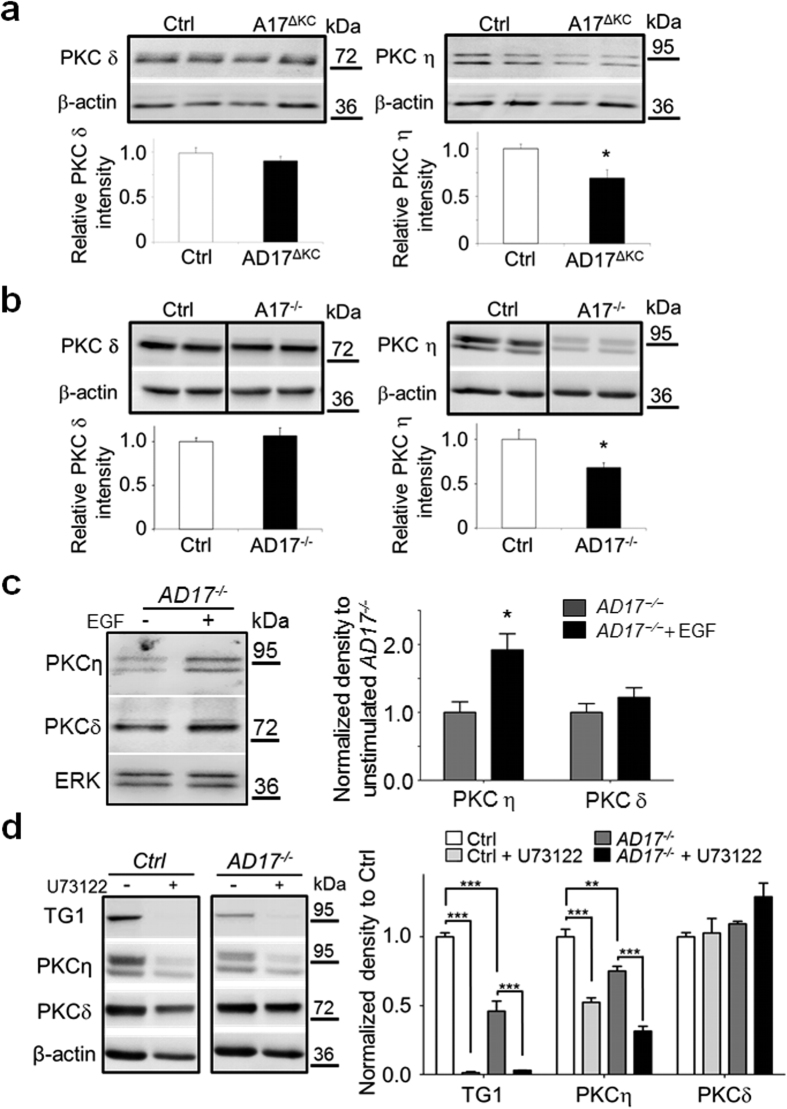
Expression of PKCη but not PKCδ is enhanced by EGFR-PLCγ1 signalling in murine epidermis. (**a**,**b**) WB analysis of (**a**) epidermal splits derived from P10 *AD17*^*∆KC*^ or wild type mice or (**b**) lysates derived from 24 h suspension cultured mouse wild type and *Adam17*^*−/−*^ keratinocytes with antibodies against PKC isoforms eta or delta, and β-actin as loading control. The graphs below show the mean ± SD of the signal intensities (n = 5), *p < 0.05; **p < 0.01. The expression of PKCη was significantly reduced in *Adam17*^*−/−*^ keratinocytes *in vivo* and *in vitro*. (**c**) *Adam17*^*−/−*^ keratinocytes were suspension-cultured on poly-HEMA with or without 40 ng/ml EGF for 24 h and further analyzed by WB for PKC isoforms eta or delta and ERK as loading control. Graph on the right shows quantitative analysis of band intensities as mean ± SEM, *p < 0.05. PKCη expression in *Adam17*^*−/−*^ keratinocytes was significantly induced by stimulation with EGF, while PKCδ expression was unchanged. (**d**) Wild type or *Adam17*^*−/−*^ keratinocytes were suspension-cultured for 24 h with or without addition of 5 μM U73122 and further analyzed by WB for TG1, PKC isoforms eta or delta. Antibodies against β-actin were used as loading control. Graph on the right shows quantitative analysis of band intensities normalized to controls (n = 3): mean ± SEM, **p < 0.01; ***p < 0.001. The supplementation of 5 μM U73122 led to dramatically decreased protein expression of PKCη and TG1 in wild type and *Adam17*^*−/−*^ keratinocytes, while the expression PKCδ was not affected. Full-length blots of (**b**) and (**d**) are presented in Supplementary Fig. S8.

The expression of three protein kinase C members, namely PKCα, PKCδ and PKCη, has been linked to epidermal terminal differentiation^36^,^37^. However, only PKCδ and PKCη seem to be involved in the induction of TG1 activity and expression^37^. To further understand which of the epidermal PKC isoforms are directly affected by absence of ADAM17/EGFR signalling, we investigated their expression in *AD17*^Δ*KC*^ versus wild type mice by WB analysis. Interestingly, we found significantly reduced expression of PKCη in *Adam17*^*−/−*^ epidermal splits as well as in ECM-disrupted differentiated *Adam17*^*−/−*^ keratinocytes. In contrast, no changes were observed for PKCδ (Fig. 5a,b) or PKCα (data not shown). The reduced PKCη expression in differentiation-committed *Adam17*^*−/−*^ keratinocytes was significantly induced by stimulation with EGF, while PKCδ expression was not affected (Fig. 5c). This result was also seen in wild type keratinocytes (Supplementary Fig. S6), indicating that PKCη expression is modulated by EGFR signalling. The linkage of PLCγ1 signalling and PKCη expression was assessed by treatment of wild type or *Adam17*^*−/−*^ keratinocyte suspensions with the PLC inhibitor U73122. Supplementation of 5 μM U73122 led to dramatically decreased protein expression of PKCη and TG1 in wild type and *Adam17*^*−/−*^ keratinocytes, while the expression PKCδ was not altered (Fig. 5d). To further confirm the role of PKCη in the modulation of TGs, wild type ECM-disrupted keratinocytes were cultivated for 24 h in the presents of selective inhibitors for either PKCδ or PKCη. The selective myristoylated pseudosubstrate inhibitor (Myr-TRKRQRAMRRRVHQING-OH) for PKCη caused a significant reduction in TG activity. In contrast, rottlerin, a selective PKCδ inhibitor, had no effect on TG activity (Supplementary Fig. S7). Taken together, these results suggest that EGFR/PLCγ1 signalling is involved in the induction of PKCη expression, which in turn leads to increased TG1 expression and activity.

### Cholesterol-sulfate supplementation restores TG activity in *Adam17* ^*−/−*^ keratinocytes *in vitro* and *in vivo*

The membrane lipid cholesterol sulfate is thought to play an important role in the physiological induction of epidermal terminal differentiation and skin barrier formation. Cholesterol sulfate is constitutively produced in the suprabasal epidermal layers where it progressively accumulates during terminal differentiation. It has previously been shown *in vitro* that stimulation of adhesive keratinocytes with cholesterol sulfate leads to increased expression of terminal differentiation markers, including TG1. This effect is mediated via PKC activity, in which cholesterol sulfate acts as a more specific activator of novel PKCs, especially PKCη^38^,^39^,^40^.

**Figure 6:**
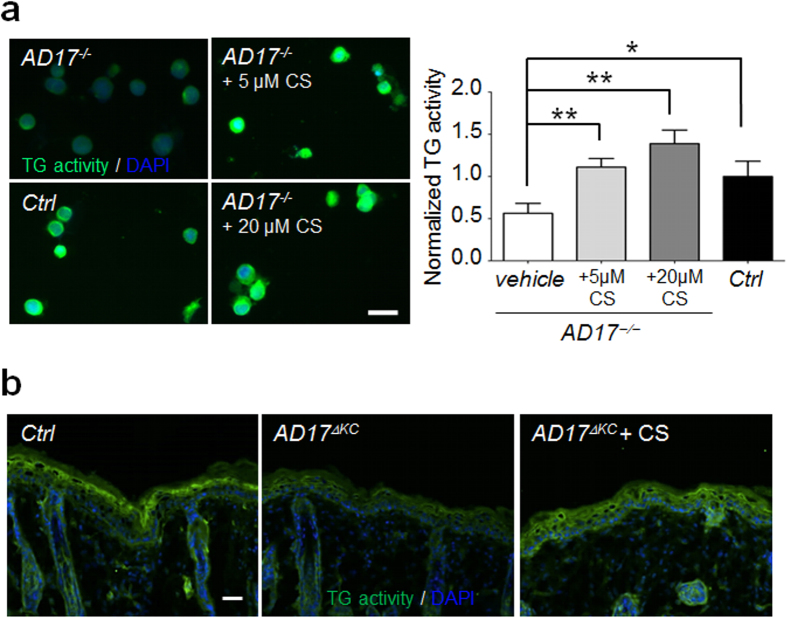
Cholesterol sulfate supplementation increased TG activity in murine keratinocytes *in vivo* and *in vitro.* (**a**) Mouse *Adam17*^*−/−*^ keratinocytes were cultured in suspension and stimulated with either 5 μM or 20 μM cholesterol-3-sultate for 24 h. Analysis of TG activity at pH 7.4 by fluorescence microscopy. TG activity of wild type keratinocytes is shown as control. n = 3. Scale bar, 20 μm. Graph shows fluorescence intensities as mean ± SEM, **p < 0.01, ***p < 0.001. Cholesterol sulfate stimulation resulted in a dose-dependent increase of TG activity in differentiating *Adam17*^*−/−*^ keratinocytes. (**b**) Cholesterol sulfate (100 μg/20 μl) or 20 μl acetone-vehicle (control) was topically applied on the shaved skin surface of 10 days old *AD17*^Δ*KC*^ mice (P10) and the skin was analyzed for *in situ* TG activity at pH 7.4 after 6 h. Untreated skin of wild type mice was used as positive control. Fluorescence microscopy analysis revealed strongly improved TG activity by cholesterol sulfate treatment (representative images of three independant experiments). Scale bar, 50 μm.

To investigate whether cholesterol sulfate can increase TG activity in *Adam17*^*−/−*^ keratinocytes, we analyzed either the addition of 5 or 20 μM cholesterol-3-sulfate to suspension cultures. Stimulation of 24 h suspension cultures completely restored the TG activity in the *Adam17*^*−/−*^ keratinocytes in a dose dependent manner (Fig. 6a). However, WB analysis revealed no increase in PKCη expression in these cells (data not shown), suggesting an induction in kinase activity. To further validate the *in vivo* relevance of our results, we treated 10 days old *AD17*^Δ*KC*^ mice by a single topically application of 100 μg cholesterol-3-sulfate or acetone vehicle (controls) on their back skin. Cholesterol sulfate treatment for 6 h was sufficient to restore the TG activity in the epidermis of *AD17*^Δ*KC*^ mice (Fig. 6b), indicating a therapeutic value of local cholesterol sulfate administration.

### Reduced TG activity in ADAM17-deficient human skin

**Figure 7:**
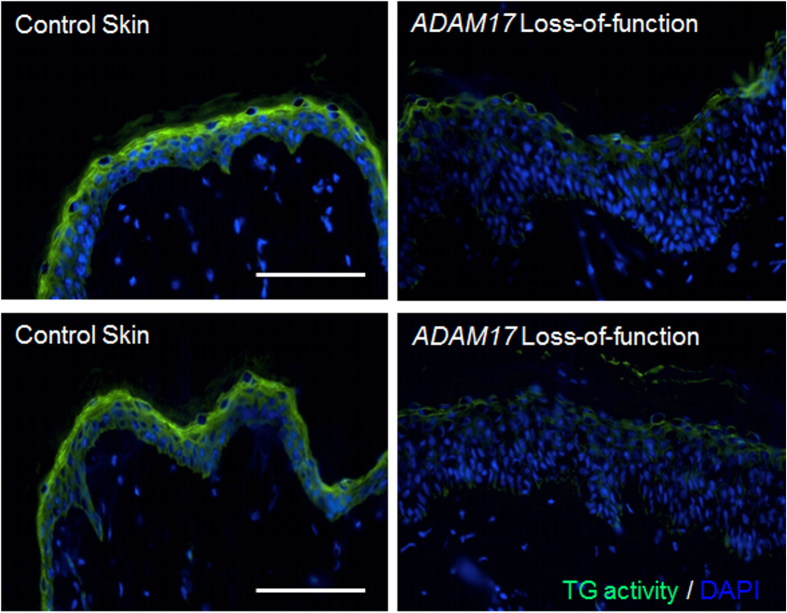
Lack of ADAM17 in humans is associated with decreased epidermal TG activity. *In situ* detection of TG activity in skin sections at pH 7.4 derived from an *ADAM17* deficient patient^24^ and healthy volunteers by fluorescence microscopy. The TG activity in the stratum granulosum and stratum corneum of the epidermis was strongly reduced in *ADAM17* deficient skin compared to control skin (representative images of three independant experiments). Scale bars, 100 μm.

After the discovery of the first two *ADAM17* deficient patients with inflammatory skin and bowel disease in 2011^24^, additional pediatric patients with deficiencies in *ADAM17* or *EGFR* were described^17^,^25^. All of these patients suffered of skin inflammation and iterated skin infections, very similar to the phenotype of keratinocyte-specific *Adam17*^*−/−*^ mice, which suggests similar skin barrier defects^13^,^23^. However, the TG activity in the skin of these patients was not evaluated. Thus, we analyzed skin cryo sections derived from an *ADAM17*-deficient patient or healthy donors by *in situ* immunofluorescence microscopy using biotinylated monodansylcadaverine at neutral pH 7.4. As shown in Fig. 7, the TG activity in the stratum granulosum and stratum corneum of the epidermis was strongly reduced in *ADAM17* deficient skin compared to control skin. Therefore, we conclude that the mechanisms of the loss of transglutaminase activity due to ADAM17-deficiency are very similar in human and mouse skin.

## Discussion

Using epidermis-specific conditional *Adam17* or *Egfr* knockout mice, we have previously shown that lack of ADAM17-dependent EGFR ligand shedding in keratinocytes leads to postnatal skin barrier defects due to decreased TG activity^13^,^26^. Here, we provide new insights into the downstream mechanisms and demonstrate that ADAM17-driven EGFR signalling directly promotes TG1 expression and activity in keratinocytes committed to differentiation mainly via PLCγ1 and PKC pathways. At first glance our results seem to be in direct conflict with previously published data, which showed EGFR-dependent repression of *TGM1* transcription in keratinocytes due to induced production of the transcription factor homeobox protein A7 (HOXA7)^41^. However, these results derived from adherent, proliferative cells that strongly express HOXA7, while its expression is lost in terminal differentiated keratinocytes due to enhanced PKC signalling^41^. Thus, our results emphasize the difference in the biological effect of EGFR signalling in delaminated terminal differentiating keratinocytes, where it promotes terminal differentiation via induction of TG1 expression and activity. In agreement, a constitutive increase of ADAM17-driven EGFR signalling was observed in tylosis (TOC) keratinocytes^42^. TOC is an autosomal dominant syndrome with focal palmoplantar keratoderma and esophageal cancer due to autosomal dominant *iRHOM2* mutations^43^. TOC skin also shows epidermal hyperproliferation and hyperkeratosis with elevated epidermal TG activity^42^.

EGFR signalling in basal membrane anchored keratinocytes promotes proliferation and survival via the ERK and Akt pathways, but not via PLCγ1^9^. In contrast, PLCγ1 signalling is proposed to lead to terminal keratinocyte differentiation through activation of PKCs^44^,^45^. Our data provides the novel finding that the ADAM17/EGFR axis in terminal differentiating keratinocytes mainly promotes PLCγ1 signalling. Although PLCγ1 activation by direct membrane recruitment to EGFR has been shown in several cell systems^46^,^47^, no such activation has been demonstrated so far in keratinocytes^48^. The increased cholesterol production and membrane integration during terminal keratinocyte differentiation seems to have an important impact on the morphological cell changes and the lipid raft-mediated signalling^49^. Therefore it is very likely that this increase in lipid microdomains leads to enriched EGFR cell surface localization in differentiation-committed keratinocytes, which in turn facilitates cell surface recruitment of PLCγ1 and EGFR/PLCγ1 signalling^50^. Upon membrane recruitment and activation, PLCγ1 catalyses the hydrolysis of phosphatidylinositol 4,5-bisphosphate (PIP_2_) to inositol 1,4,5-trisphosphate (IP_3_) and diacylglycerol (DAG), which both act as second messengers in the activation of PKCs in terminal differentiation^44^. Thus, our data suggest that EGFR signalling stabilizes terminal differentiation by maintenance of PKC activity in differentiation-committed keratinocytes via PLCγ1 activation.

*In vitro* studies revealed that lack of EGFR/ERK signalling in suspension cultured keratinocytes for more than 24 h can lead to apoptosis due to the loss of basement membrane interactions^30^, which could be the reason for decreased activity and expression of TG1 in *Adam17*^*−/−*^ keratinocytes. Although we used a different suspension culture system than the authors and cultured the cells for a maximum of 24 h under EGF supplementation, we saw reduced ERK activation in *Adam17*^*−/−*^ keratinocytes. However, we neither detected decreased ERK signalling nor increased apoptosis as determined by TUNEL labelling in the epidermis of *AD17*^Δ*KC*^ mice, which questions the *in vivo* relevance of the above findings. Furthermore, it has been demonstrated that PKCs can be involved in the activation of ERK signalling^51^,^52^. Thus, reduced PKC signalling might cause decreased ERK activation in *Adam17*^*−/−*^ keratinocytes.

Our results suggest that ADAM17-driven EGFR signalling in differentiated keratinocytes acts in two different ways to induce PKC signalling and promote TG1 activity. Firstly, it activates PLCγ1 that produces second messengers for the activation of keratinocyte-derived PKC isoforms, namely the classical calcium- and diacylglycerol-dependent PKCα and the novel calcium-independent but diacylglycerol-dependent PKCδ, PKCε and PKCη^31^. Secondly, it selectively induces the expression of PKCη. This result is of particular interest, since only little is known about the transcriptional control of the novel PKCs during terminal differentiation. The induction of PKCη expression is probably driven via EGFR/PLCγ1 signalling, since PKCη expression was highly sensitive to PLCγ1 inhibition. However, it can not be ruled out that other signalling pathways are involved as well. PKCη is strongly linked to terminal keratinocyte differentiation, since it is selectively expressed in suprabasal epidermal layers and its viral overexpression in adherent human keratinocytes caused growth inhibition and induction of TG1 expression and activity^37^,^39^,^53^. However, PKCη activity seems not to be crucial for epidermal differentiation in normal skin and is most likely replaceable by other PKC isoforms, since PKCη null mice showed no defects in skin architecture and development^54^.

Taken together, our data suggest ADAM17/EGFR-driven PLCγ1 and PKC pathways as important promoters of TG1 expression during terminal keratinocyte differentiation and skin barrier formation. It further provides evidence that topical skin application of TPA or cholesterol sulfate can restore the TG activity in the epidermis of *AD17*^Δ*KC*^ mice, which should have a beneficial effect on the skin barrier function. In contrast to the tumor-promoting phorbol ester TPA, that activates almost all PKCs, cholesterol sulfate is a more specific activator of novel PKCs, especially PKCη, and rather acts as an anticarcinogenic component^38^,^39^,^40^. Thus, cholesterol sulfate seems to have therapeutic value for the local treatment of disrupted EGFR signalling caused skin barrier defects in atopic dermatitis^55^ or EGFR inhibitor-treated cancer patients^18^.

## Methods

### Material

The PI3K inhibitor Ly294002 was purchased from Merck Millipore (Germany) and the ERK1/2 inhibitor PD98059 derived from Cell signalling technology (Germany). The general PKC inhibitors Bisindolylmaleimide and Gö6983 were obtained from Sigma-Aldrich (Germany) and Merck Millipore (Germany), respectively. Rottlerin, a selective inhibitor of PKCδ was obtained from Sigma-Aldrich (Germany) and the PKCη pseudosubstrate inhibitor, myristoylated (Myr-TRKRQRAMRRRVHQING-OH) was bought from Merck Millipore. Recombinant murine EGF, human TGF-α and human Epiregulin were obtained from Peprotech (Germany).

### Animals

The generation of *Adam17*^*flox/flox*^ Krt14-Cre (*AD17*^Δ*KC*^) and *Egfr*^*flox/flox*^ Krt14-Cre (*Egfr*^Δ*KC*^) mice has been described previously^13^. All mice were of mixed genetic background (129 Sv, C57BL/6), and all comparisons were between littermates. In experiments, mice received a single 100 μl application of either 1.5 μg 12-O-tetradecanoylphorbol-13-acetate (TPA, Sigma-Aldrich) or 100 μg cholesterol 3-sulfate (Sigma-Aldrich) in acetone or acetone vehicle on the shaved back skin. The mice were maintained in the Center for Experimental Models and Transgenic Service (CEMT) of the Medical Center University of Freiburg, and all experiments were performed according to the guidelines of the German Animal Welfare association and approved by the Regierungspräsidium Freiburg (G-11/98 and G-15/145).

### Primary keratinocyte preparation and cultivation

Keratinocytes were isolated from the skin of neonatal *AD17*^Δ*KC*^ mice and their wild type littermates as described previously^56^. The primary cells were cultured in defined serum-free keratinocyte growth medium (CellnTec, Switzerland) supplemented with 100 U/ml penicillin, 100 μg/ml streptomycin (Invitrogene). The cells were maintained at 37 °C, 5% CO_2_ and 95% humidity. Keratinocytes derived from passages 1–2 were used for the experiments.

### Keratinocyte suspension culture

Suspension culture on poly (2-hydroxyethyl methacrylate (HEMA))–coated plates was performed as previously described^10^. 6-well or 12-well plates were coated with either 10 mg or 4 mg poly-HEMA (Sigma-Aldrich) respectively, followed by extensive PBS washes. 1 × 10^6^ or 0,5 × 10^6^ cells in keratinocyte growth medium (CellnTec, Switzerland) were added to each coated 6-well or 12-well respectively and incubated in a humidified incubator with 5% CO2 in air at 37 °C for 24 h. EGFR stimulations were performed with either 30 ng/ml EGF, TGFα or Epiregulin. In some experiments, the cell suspensions were supplied with 500 nM GÖ 6983, 1 μM bisindolylmaleimide, 50 μM Ly294002, 50 μM PD98059, or 5 μM U73122 for 24 h. Cell suspensions were harvested by centrifugation and either used for preparation of cell lysates or detection of TG activity. For *in vitro* TG activity detection, 24-h suspension-cultured keratinocytes were attached on gelatin-coated coverslips for 1 h and TG activity was determined as described below.

### Quantitative RT-PCR analysis

Total RNA from suspension-cultured *Adam17*^*−/−*^ and wild type keratinocytes was extracted using RNeasy kit (Qiagen). 1 μg of total RNA was reverse transcribed using a First Strand cDNA Synthesis kit (Fermentas). Relative quantification of gene expression was performed by real-time quantitative PCR using iQ SYBR-Green Supermix on the CFX96TM C1000TM Thermal Cycler (Bio-Rad, Germany) following the manufacturer’s protocols. The used primer sequences for *Tgm1*, *Adam17*, *Egfr, Inv*, *Krt14* and *Gapdh* were described previously^13^. Relative expression was normalized for levels of *Gapdh*.

### Transglutaminase activity assay

For *in situ* detection of TG activity in skin sections or cells, the biotinylated amine donor substrate monodansylcadaverine (MDC) was used as described earlier^13^. 5 μm thick cryostat sections were air dried and preincubated with 1% BSA in 0.1 M Tris-HCl, pH 7.4 for 30 min at room temperature. The sections were then incubated for 45 to 60 min with 100 μM MDC, 5 mM CaCl_2_, 0.1 M Tris-HCl, pH 7.4. After stopping the reaction with 10 mM EDTA and extensive PBS washing, the sections were stained with Streptavidin-conjugated Alexa Fluor 488 (Invitrogen) and DAPI-supplemented mounting medium.

### Immunoblotting

For WB analysis dispase-separated epidermis splits or cells were homogenized in 50 mM Tris-HCl, pH 8.0, 0.15 M NaCl, 1% Nonidet P-40, 0.5% sodium deoxycholate supplemented with 2 mM EDTA, 5 mM 1,10- ortho-phenanthroline (Sigma-Aldrich) and protease inhibitor cocktail set III (Calbiochem) as described previously^13^. Total protein content was determined using the BCA^TM^ protein assay kit (Invitrogen). 30 μg of protein was separated and transferred either onto nitrocellulose or PVDF membranes. Protein detection on membranes was performed with subsequent primary antibodies: rabbit anti-PKCη, rabbit anti PKCδ, rabbit anti-transglutaminase 1 (TG1), (Santa Cruz Biotechnology), rabbit anti-PLCγ1, rabbit anti-phospho PLC-γ1, rabbit anti-EGFR (Cell Signaling), rabbit anti–phospho EGFR (pY1068, clone EP774Y; Epitomics Inc.), rabbit anti-ERK1/2, mouse anti-β-actin (Sigma-Aldrich) and mouse anti-GAPDH (Invitrogen). Visualization was performed with secondary horseradish peroxidase labeled goat anti-mouse IgG (Merck) or horseradish peroxidase labeled goat anti-rabbit IgG (KPL) and Amersham ECL prime western blotting detection reagent as described by the manufacturer (GE Healthcare).

### TUNEL assay

The fluorometric DeadEnd™TUNEL System (Promega) was used to analyze apoptotisis in paraffin skin sections (5 μm) of *AD17*^Δ*KC*^ mice and their wild type littermates according the manufacturer´s recommendations.

### Patient material

Skin samples were obtained from a 17-year-old male *ADAM17*-deficient patient and healthy volunteers undergoing cosmetic surgery after informed consent and in adherence to the declaration of Helsinki principles. Clinical details of the *ADAM17*-deficient patient were described elsewhere^24^. The study was approved by the ethics committee of the Queen Mary University of London.

### Statistics

The data are present as mean ± SEM. Data of two groups were analyzed for significance using the unpaired Student´s t test and differences are considered to be statistically significant at P < 0.05.

## Additional Information

**How to cite this article**: Wolf, C. *et al*. ADAM17/EGFR axis promotes transglutaminase-dependent skin barrier formation through phospholipase C γ1 and protein kinase C pathways. *Sci. Rep.* **6**, 39780; doi: 10.1038/srep39780 (2016).

**Publisher's note:** Springer Nature remains neutral with regard to jurisdictional claims in published maps and institutional affiliations.

## Supplementary Material

Supplementary Material
