# Corrigendum: ADAM17/EGFR axis promotes transglutaminase-dependent skin barrier formation through phospholipase C γ1 and protein kinase C pathways

**DOI:** 10.1038/srep41343

**Published:** 2017-01-23

**Authors:** Cristina Wolf, Yawen Qian, Matthew A. Brooke, David P. Kelsell, Claus-Werner Franzke

Scientific Reports 6: Article number: 3978010.1038/srep39780; published online: 12 22 2016; updated: 01 23 2017

The original version of this Article contained an error in the title of the paper, where the word “phospholipase” was incorrectly given as “phosholipase”. This has now been corrected in the PDF and HTML versions of the Article.

