# Supplementary material for: ADAM17/EGFR axis promotes transglutaminase-dependent skin barrier formation through phospholipase C γ1 and protein kinase C pathways
Source: Sci Rep. 2016 Dec 22;6:39780. doi: 10.1038/srep39780 (PMC5177948; doi:10.1038/srep39780)
Supplement: Supplementary Material [file srep39780-s1.pdf]

Supplementary material for:

ADAM17/EGFR axis promotes transglutaminase-dependent skin barrier formation through phospholipase C γ1 and protein kinase C pathways.

Cristina Wolf[1, 3, +], Yawen Qian[1, +], Matthew A. Brooke[2], David P. Kelsell[2], Claus-Werner Franzke[1,#]

[1] Department of Dermatology, Medical Center - University of Freiburg, Freiburg, Germany

[2] Blizard Institute, Barts and the London School of Medicine and Dentistry, Queen Mary University of London, London, United Kingdom

[3] present address: Luxembourg Centre for Systems Biomedicine, University of Luxembourg, Luxembourg

[+] contributed equally

[#] Corresponding author address: Dept. of Dermatology, Medical Center - University of Freiburg, Hauptstrasse 7, 79104 Freiburg, Germany, Tel: +49 761 27067850, Fax: +49 761 27067200; Email: claus-werner.franzke@uniklinik-freiburg.de

Supplementary Figures:

Figure S1 - Figure S8

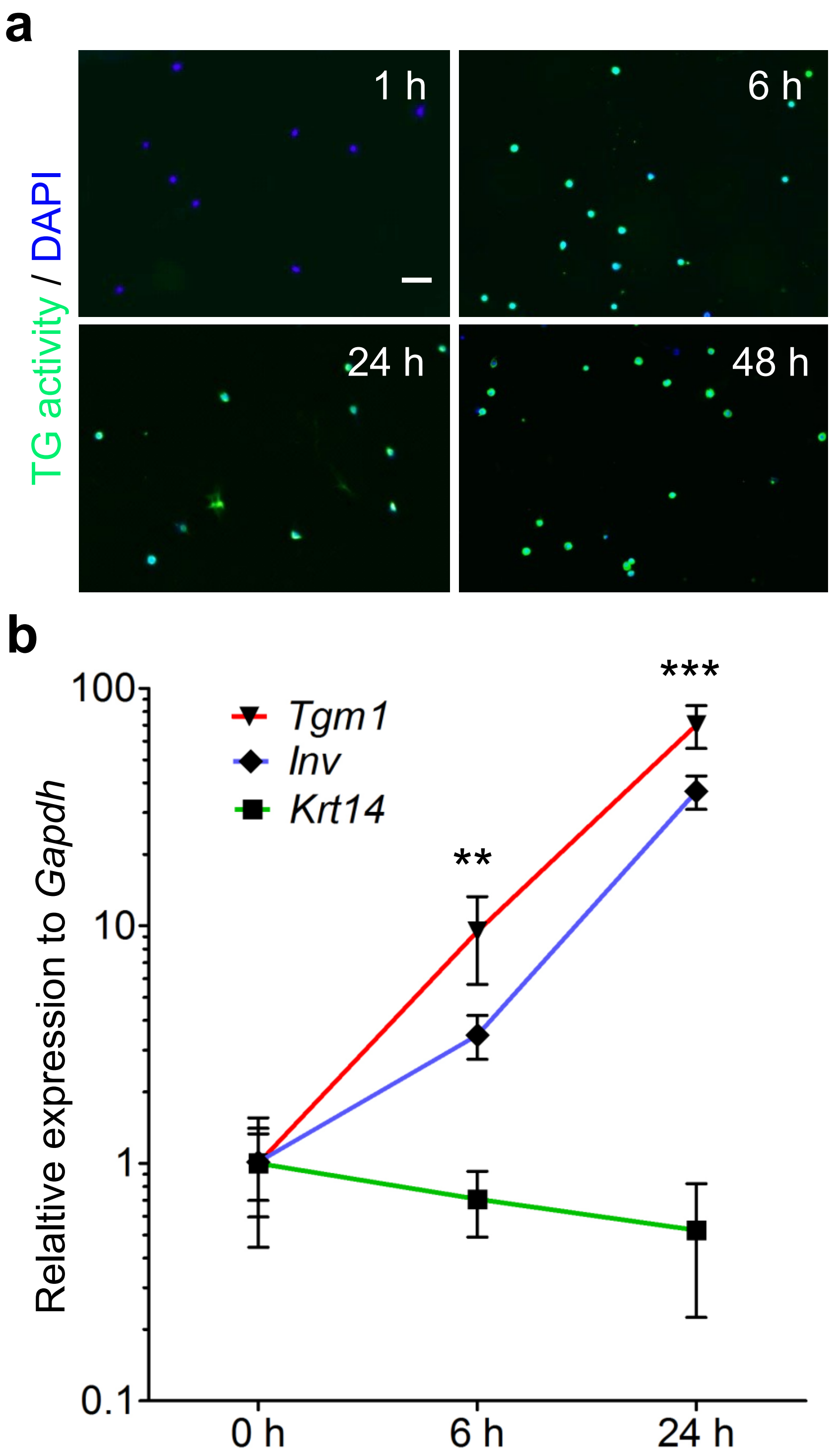

Figure S1. TG activity and gene expression in murine wild type keratinocytes during suspension culture. (**a** and **b**) ECM-disrupted wild type keratinocyte suspensions were cultured for up to 48 h and either analyzed by (**a**) immunfluorescence for TG activity or (**b**) qPCR from total RNA for gene expression of *Tgm1*, *Inv*, and *Krt14* at indicated times. *Gapdh* was used as housekeeping gene. n=3. Data as mean ± SD, ** p<0.01, ***p<0.001.

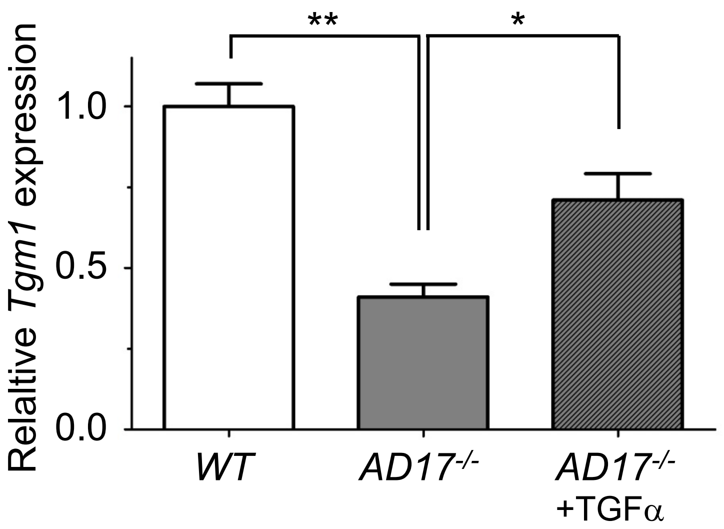

Figure S2. *Tgm1* gene activity in murine Adam17$^{-/-}$ keratinocytes is induced by TGF-α. *Adam17$^{-/-}$* keratinocytes were cultured with or without addition of 40 ng/ml TGF-α in suspension on poly-HEMA for 24 h. Untreated wild type keratinocytes were used as control. Total RNA of the cells was further analyzed for *Tgm1* transcription by qPCR. *Gapdh* was used as housekeeping gene. Addition of TGF-α significantly stimulates *Tgm1* transcription in *Adam17$^{-/-}$* keratinocytes. n=3. Data as mean ± SD, *$p<0.05$, **$p<0.01$.

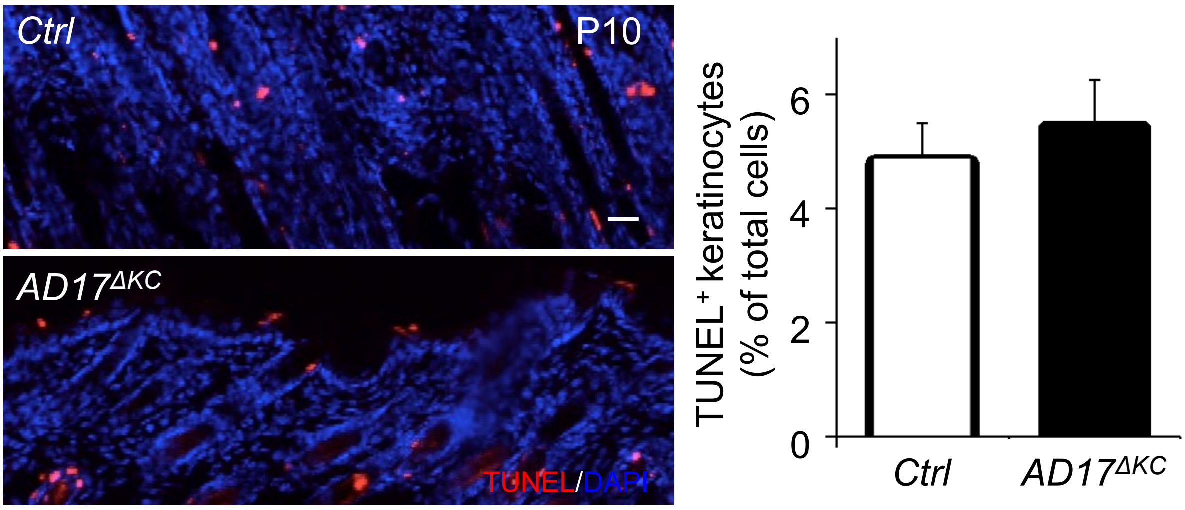

Figure S3. Lack of ADAM17 in keratinocytes does not lead to increased epidermal apoptosis in mouse skin. Skin paraffin sections of 10 days old *AD17*$^{\Delta KC}$ and wild type mice were analysed by TUNEL labelling as described in the methods. No difference in the number of TUNEL^{+} keratinocytes were detected in *A17*$^{\Delta KC}$ skin versus control skin. n=3. Data as mean ± SD.

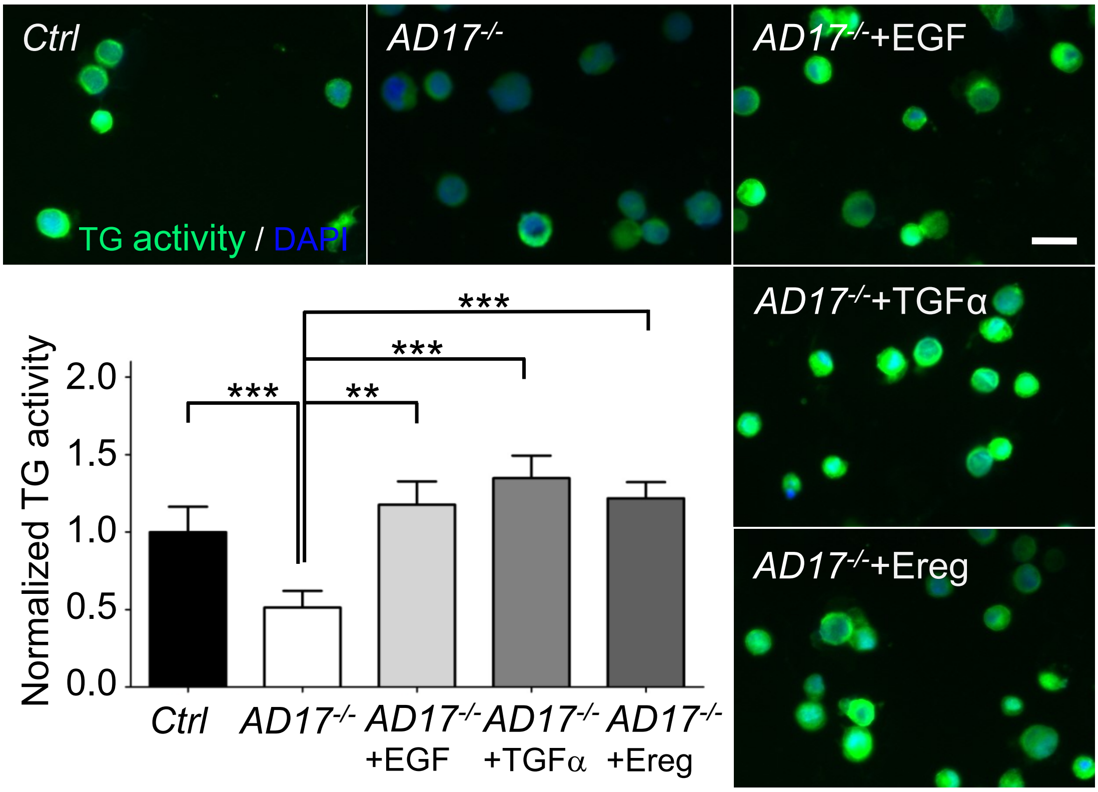

Figure S4. TG activity in murine *Adam17*$^{-/-}$ keratinocytes is responsive to EGFR-ligands. *Adam17*$^{-/-}$ keratinocytes were cultured in suspension and stimulated with either 30 ng/ml EGF, TGFα or Epiregulin for 24 h. Analysis of TG activity was done by immunofluorescence. TG activity of wild type keratinocytes is shown as control. n=3. Data as mean ± SEM, ** $p<0.01$, ***$p<0.001$.

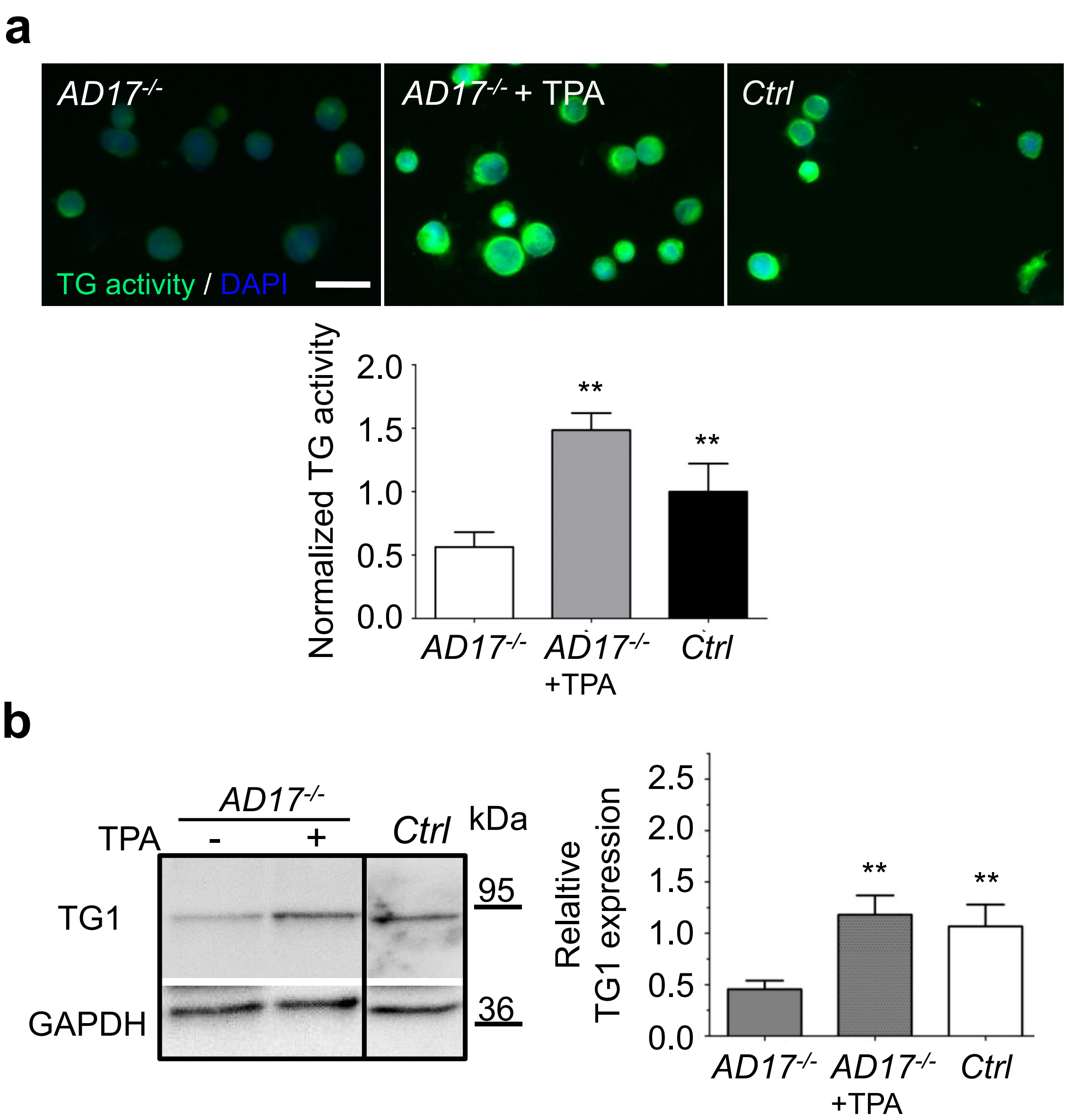

Figure S5. The phorbol ester TPA restores TG1 expression in murine *Adam17*$^{-/-}$ keratinocytes. (**a** and **b**) *Adam17*$^{-/-}$ keratinocyte suspension cultures were precultured for 24 h. Afterwards, 1 µM TPA or acetone vehicle were added to the cells and they were incubated for another 6 h and analyzed for (**a**) *in situ* TG activity by fluorescence microscopy (n=3) and (**b**) TG1 expression by WB analysis (n=3). As control, wild type keratinocytes were cultured in parallel. GAPDH was used as loading control. *In vitro* stimulation of differentiation-committed *Adam17*$^{-/-}$ keratinocytes with TPA for 6 h leads to increased TG1 activity and expression. Data as mean ± SEM, n=3, ** $p<0.01$.

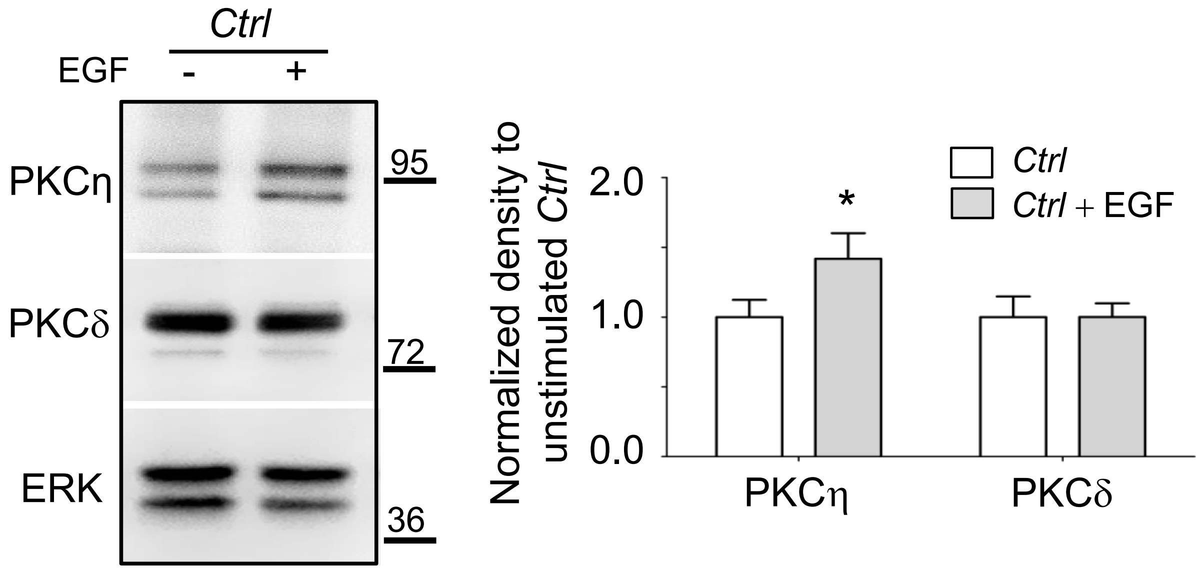

Figure S6. Expression of PKCη in mouse wild type keratinocytes is inducible by EGF supplementation. Wild type keratinocytes were suspension-cultured on poly-HEMA with or without 40 ng/ml EGF for 24 h and further analyzed by WB for PKC isoforms η or δ and ERK as loading control. Graph on the right shows quantitative analysis of band intensities: mean ± SEM, n=3, *p<0.05. PKCη expression was significantly induced by EGF stimulation, while PKCδ expression was unchanged.

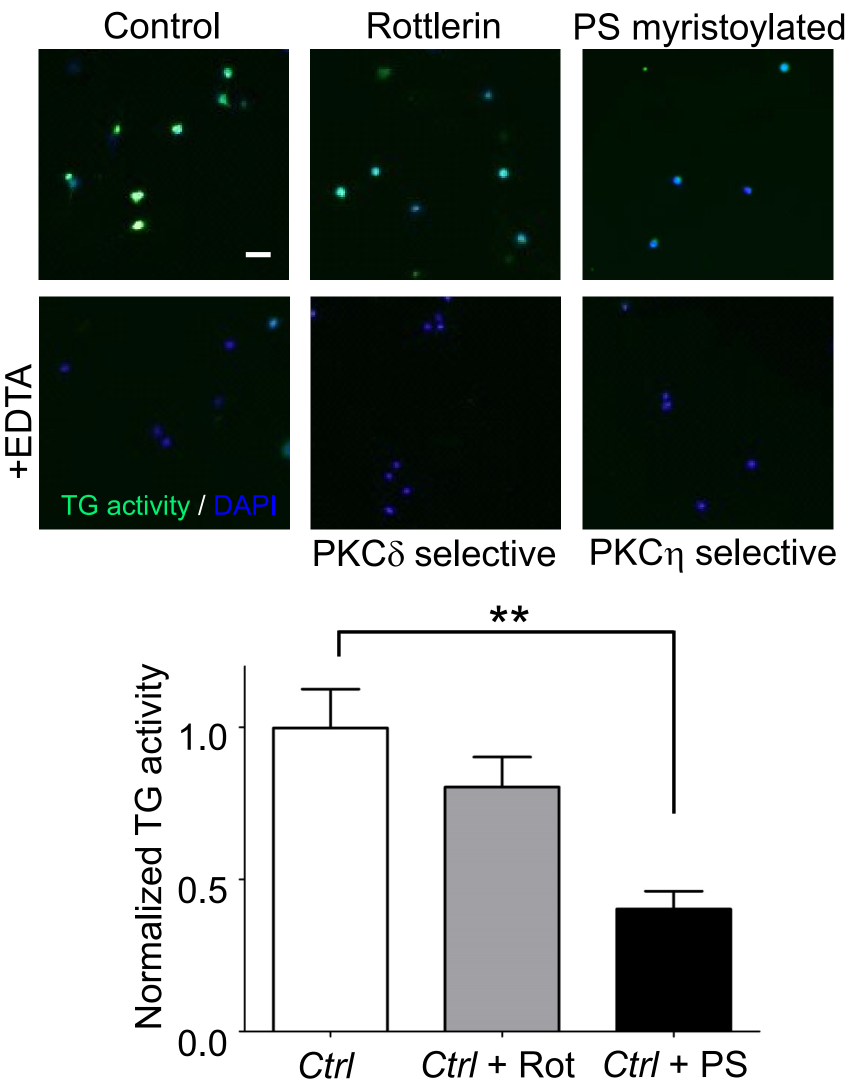

Figure S7. Selective inhibition of PKCη, but not of PKCδ significanly reduces TG activity in differentiating mouse keratinocytes. Wild type keratinocytes were suspension-cultured on poly-HEMA for 24 h with either DMSO (vehicle control), 1 μM of myristoylated pseudosubstrate (PS) inhibitor of PKCη or 6 μM of Rottlerin, a selective polyphenol inhibitor of PKCδ and analyzed for *in situ* TG activity by fluorescence microscopy. Graph below shows quantitative analysis of fluorescence intensities: mean ± SEM, n=3, **$p<0.01$. The selective PKCη inhibitor caused significant reduction of TG activity, while selective PKCδ inhibition had no effect.

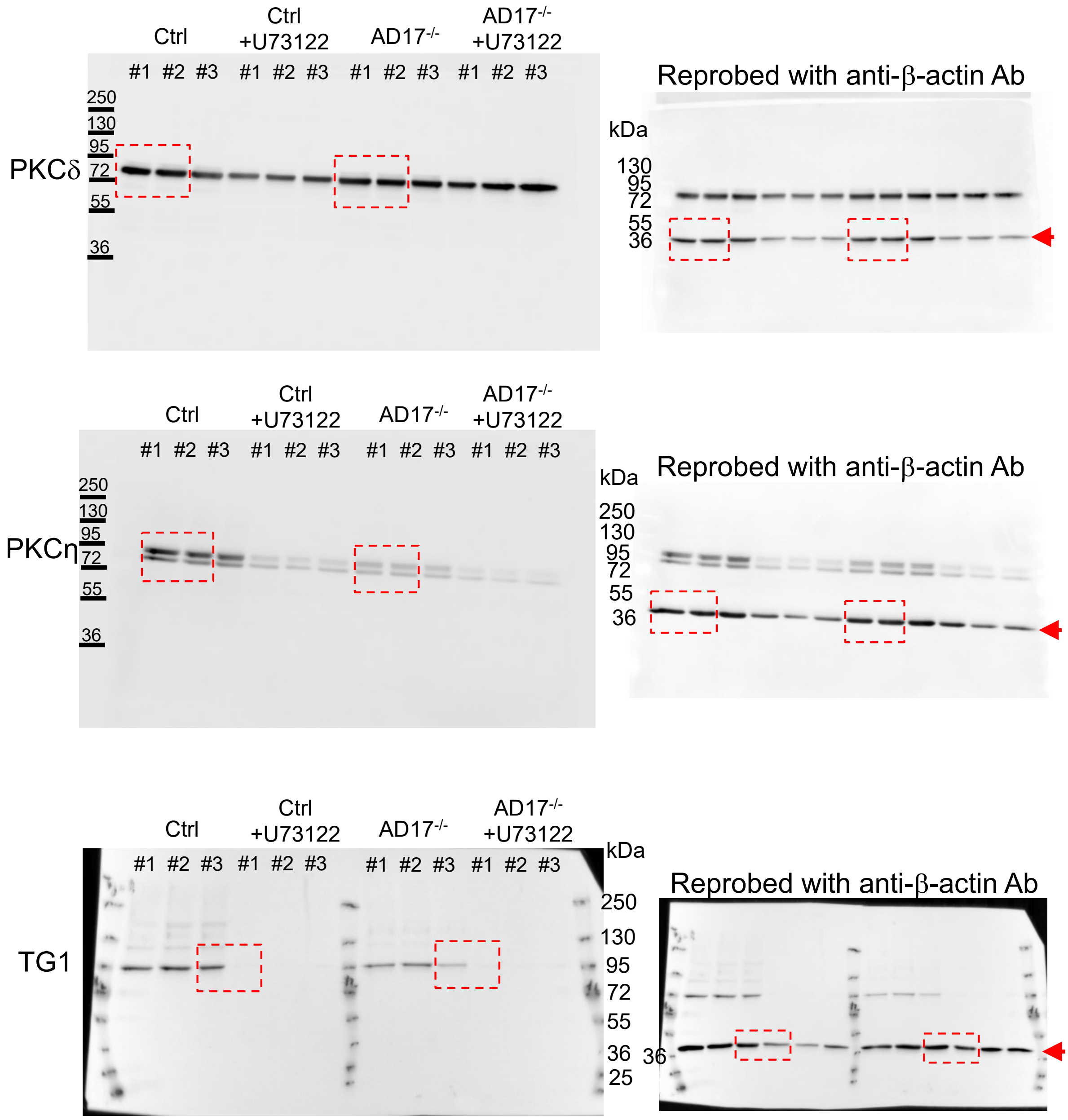

Figure S8. Full-length WBs for cropped blots shown in Figure 5 b & d. Wild type or *Adam17*$^{-/-}$ keratinocytes were suspension-cultured for 24 h with or without addition of 5 µM U73122 and further analyzed by WB for TG1, PKC isoforms eta or delta. Antibodies against β-actin were used to detect loading. n=3. Red dashed retangles indicate cropped blots.
